# Supplementary material for: Identification of Key Aroma Compounds Responsible for the Floral Ascents of Green and Black Teas from Different Tea Cultivars
Source: Molecules. 2022 Apr 28;27(9):2809. doi: 10.3390/molecules27092809 (PMC9100887; doi:10.3390/molecules27092809)
Supplement: Supplementary file 1 [file molecules-27-02809-s001.zip › molecules-1695276-supplementary.pdf]

Table S1 Comprehensive sensory evaluation results of green and black teas.

Tea samples	Dry tea appearance		Tea infusion color		Tea taste		Tea aroma		Brewed leaves		Total score [a]
	Description	Score	Description	Score	Description	Score	Description	Score	Description	Score	
Green tea											
Chunyu2	Burly, greyish green, hairy	92	light yellowish green, bright	92	*Umami*, mellow	94	**Long-lasting lily of the valley fragrance**	95	Burly, delicate green, comparatively bright	93	93.45
Chunyu1	Delicate green, comparatively smooth	93	Delicate green, bright	93	*Umami*, mellow	93	Faint scent	91	Slightly thin, delicate green, comparatively bright	91.5	92.35
Longjing43	Greyish green, hairy	90	Yellowish green, bright	91	Astringent	87	Green and grassy	88	Green, bright	90	88.70
Zhenong117	Burly, barely delicate green, barely smooth	93	Light green, bright	94	*Umami*, barely mellow, smooth	94	Tender, strong aroma	93	Burly, delicate green, bright	94	93.50
Jinguanyin	Slightly flat and burly, dark green	89	Bright yellow	92	Barely *umami*, mellow	92	**Long-lasting rose fragrance**	93	Burly, delicate green, bright	94	91.70
Black tea											
Chunyu2	Tight and sturdy, hairy, dark and smooth	93	Light orange red, barely bright	92	*Umami*, mellow	93	**Floral aroma**	93	Burly, red, comparatively bright	91	92.70
Chunyu1	Tight and slender, hairy, dark and smooth	94	Orange red, comparatively bright	93	Barely mellow and thick	92	Sweet aroma	92	Slightly slender, red, comparatively bright	91	92.50
Longjing43	Tight and slender, a bit hairy, dark and barely smooth	91	Light orange red, comparatively bright	89	Light, comparatively thin	86	Sweet aroma	86	Red, comparatively dark	89	87.85
Zhenong117	Tight and slightly sturdy, hairy, dark and smooth	93	Light orange red, comparatively bright	92	Barely mellow, slightly astringent	89	Faint sweet aroma, a bit green	88	Burly, comparatively red and bright	92	90.35
Jinguanyin	Tight and sturdy, hairy, dark and smooth	92	Dark orange yellow, barely bright	91	Barely mellow	91	**Long-lasting rose fragrance, sweet aroma**	95	Burly, comparatively red and bright	92	92.35

Total score= The score of dry tea appearance×25%+ the score of tea infusion color×10%+the score of tea taste×30%+the score of tea aroma×25%+the score of brewed leaves×10%.

Table S2 The volatile compositions of green teas prepared from different tea cultivars (μg guaiacol equivalent/g dry tea).[a]

Compounds	RI [b]	Chunyu2	Chunyu1	Longjing43	Zhenong117	Jinguanyin
Alcohols						
3,7-Dimethylocta-1,6-dien-3-ol	1524	64.79±1.33	10.07±1.09	6.77±1.17	20.73±1.73	10.77±0.34
(2*E*)-3,7-Dimethylocta-2,6-dien-1-ol	1806	11.32±0.37	7.50±0.84	14.08±2.21	3.51±0.43	31.13±1.47
2-[(2*S*,5*S*)-5-Ethenyl-5-methyloxolan-2-yl]propan-2-ol	1443	4.01±0.91	ND	0.79±0.25	0.41±0.22	1.03±0.12
(*Z*)-hex-3-en-1-ol	1370	1.29±0.16	0.47±0.04	0.77±0.11	1.51±0.20	0.24±0.03
dodecan-1-ol	1868	1.35±0.24	0.66±0.16	0.63±0.31	0.44±0.07	0.62±0.07
(1*S*,2*R*,5*S*,7*R*,8*R*)-2,6,6,8-Tetramethyltricyclo[5.3.1.01,5]undecan-8-ol	2022	0.99±0.29	0.48±0.03	0.67±0.59	0.41±0.06	0.46±0.15
2-(4-Methylcyclohex-3-en-1-yl)propan-2-ol	1661	1.90±0.21	ND	ND	ND	0.41±0.09
Pentan-1-ol	1242	ND	0.62±0.03	0.38±0.08	0.46±0.06	0.25±0.04
Oct-1-en-3-ol	1428	0.19±0.02	0.41±0.03	0.68±0.17	ND	0.43±0.01
Octan-1-ol	1533	ND	0.66±0.06	1.01±0.31	ND	ND
2-Ethylhexan-1-ol	1465	0.28±0.07	0.26±0.06	0.46±0.11	0.43±0.14	0.20±0.03
6-Ethenyl-2,2,6-trimethyloxan-3-ol	1704	1.28±0.24	ND	ND	ND	0.20±0.01
Nonan-1-ol	1628	0.21±0.02	0.60±0.15	0.25±0.04	0.22±0.02	0.13±0.02
(5*E*)-3,7-Dimethylocta-1,5,7-trien-3-ol	1582	0.43±0.08	0.15±0.02	0.05±0.01	0.34±0.05	0.26±0.02
(*E*)-Oct-2-en-1-ol	1586	0.16±0.02	ND	0.24±0.03	0.22±0.02	0.30±0.03
(*E*)-3,7,11,15-Tetramethylhexadec-2-en-1-ol	1871	0.18±0.02	ND	0.39±0.05	ND	0.28±0.06
2-Phenylethanol	1838	0.37±0.04	ND	0.14±0.03	0.09±0.04	0.22±0.02
(*E*)-Hex-2-en-1-ol	1390	0.36±0.16	ND	ND	0.33±0.07	ND
2-[(1*S*)-4-Methylcyclohex-3-en-1-yl]propan-2-ol	1664	ND	0.16±0.05	ND	0.50±0.10	ND
Tetradecan-1-ol	2041	ND	ND	ND	ND	0.62±0.19
(1*S*,4*R*)-1,6-Dimethyl-4-propan-2-yl-3,4,4a,7,8,8a-hexahydro-2H-naphthalen-1-ol	2044	0.19±0.03	ND	ND	ND	0.42±0.24
Phenylmethanol	1821	0.21±0.08	ND	ND	ND	0.40±0.11
Hexan-1-ol	1342	0.13±0.01	0.21±0.01	0.09±0.02	0.18±0.01	ND
Heptan-1-ol	1436	ND	0.22±0.06	0.16±0.04	0.17±0.02	ND
Heptan-2-ol	1310	0.50±0.08	ND	ND	ND	ND
(*Z*)-Non-3-en-1-ol	1650	0.07±0.01	ND	0.09±0.03	0.05±0.01	ND
Total alcohols		90.21±2.99	22.45±2.07	27.66±2.82	29.97±2.77	48.34±2.15

Aldehydes						
2,4-Dimethylbenzaldehyde	1765	5.55±0.82	3.46±0.53	8.64±1.03	4.44±0.52	4.87±0.23
Pentanal	961	0.49±0.15	1.76±0.07	1.17±0.23	1.07±0.33	0.51±0.07
3-Methylbutanal	936	0.08±0.01	1.59±0.20	0.56±0.19	0.92±0.11	0.31±0.19
Hexanal	1044	0.63±0.01	ND	1.24±0.35	1.13±0.20	0.41±0.14
(*Z*)-Hept-2-enal	1289	0.46±0.06	0.47±0.18	0.41±0.05	0.71±0.24	0.47±0.12
Nonanal	1348	0.41±0.06	0.42±0.02	0.99±0.38	0.31±0.07	0.22±0.05
Heptanal	1141	ND	0.98±0.29	0.58±0.14	0.56±0.12	ND
Benzaldehyde	1487	0.28±0.13	0.24±0.01	0.56±0.15	0.16±0.01	0.14±0.01
(2*E*)-3,7-Dimethylocta-2,6-dienal	1690	0.36±0.02	ND	ND	ND	0.77±0.03
(*E*)-Oct-2-enal	1396	ND	ND	0.18±0.05	0.22±0.01	0.22±0.04
Decanal	1452	0.56±0.12	ND	ND	ND	ND
(2*Z*)-3,7-Dimethylocta-2,6-dienal	1643	ND	ND	0.06±0.01	ND	0.09±0.01
Total aldehydes		8.81±1.06	8.93±0.80	14.40±1.23	9.52±0.95	8.01±0.39
Ketones						
Octane-2,3-dione	1290	0.65±0.15	0.62±0.05	0.84±0.27	0.72±0.15	0.73±0.03
6,10,14-Trimethylpentadecan-2-one	2027	0.55±0.11	0.16±0.03	0.35±0.08	0.40±0.03	1.72±0.54
3-Methyl-2-[(*Z*)-pent-2-enyl]cyclopent-2-en-1-one	1850	0.55±0.03	0.33±0.05	0.35±0.06	0.89±0.13	0.53±0.06
4-Methylpent-3-en-2-one	1108	ND	1.32±0.10	0.16±0.02	0.27±0.02	0.40±0.06
(*E*)-4-(2,6,6-Trimethylcyclohexen-1-yl)but-3-en-2-one	1846	0.18±0.03	ND	0.35±0.05	0.14±0.01	ND
6-Methylhept-5-en-2-one	1311	ND	ND	0.28±0.09	0.17±0.01	0.17±0.05
Oct-1-en-3-one	1272	ND	ND	0.16±0.06	0.15±0.02	0.17±0.06
Total ketones		1.93±0.14	2.42±0.13	2.49±0.45	2.74±0.34	3.72±0.57
Esters						
Bis(2-methylpropyl) benzene-1,2-dicarboxylate	2118	3.04±0.81	1.96±0.24	2.28±0.08	2.54±0.51	1.79±0.27
Diethyl benzene-1,2-dicarboxylate	2084	8.44±3.11	ND	ND	ND	ND
[(*Z*)-hex-3-enyl] acetate	1279	1.49±0.20	0.33±0.11	0.41±0.12	3.25±0.56	0.27±0.01
[(*Z*)-hex-3-enyl] butanoate	1416	1.16±0.05	ND	0.22±0.06	1.79±0.09	0.39±0.02
Methyl 2-hydroxybenzoate	1727	0.47±0.03	0.49±0.18	0.95±0.13	0.81±0.19	0.29±0.03
1-*O*-Butyl 2-*O*-octyl benzene-1,2-dicarboxylate	2032	0.84±0.31	ND	0.56±0.14	ND	0.46±0.09
Methyl hexadecanoate	2051	0.36±0.18	0.21±0.05	0.29±0.03	0.19±0.02	0.36±0.08
Methyl 2-methyl-3-oxobutanoate	1669	0.92±0.17	ND	ND	0.22±0.05	0.24±0.03
[(*Z*)-hex-3-enyl] hexanoate	1609	0.41±0.06	0.21±0.05	ND	0.09±0.02	ND
Propan-2-yl tetradecanoate	1897	0.20±0.09	0.09±0.01	0.14±0.03	0.17±0.07	0.11±0.02
Dibutyl benzene-1,2-dicarboxylate	2033	ND	ND	ND	0.60±0.08	ND

Total esters		17.34±2.77	3.28±0.15	4.86±0.37	9.67±1.14	3.91±0.23
Pyrroles						
1-Ethylpyrrole-2-carbaldehyde	1571	0.12±0.01	0.23±0.06	0.58±0.05	0.22±0.02	ND
Miscellaneous						
Methylsulfanylmethane	899	1.17±0.15	8.19±0.74	0.50±0.16	4.31±0.76	4.32±0.66
Naphthalene	1684	3.83±0.94	2.45±0.75	4.27±0.18	4.21±0.39	3.13±0.13
2,4-Ditert-butylphenol	2071	2.56±0.07	0.79±0.37	3.91±0.78	1.06±0.14	2.88±1.23
7-Methyl-3-methylideneocta-1,6-diene	1114	2.79±0.42	0.97±0.17	1.08±0.02	0.84±0.15	2.52±0.10
Methyl (*Z*)-N-hydroxybenzenecarboximidate	1750	1.39±0.15	ND	0.68±0.28	0.54±0.04	1.57±0.19
(3*E*)-3,7-Dimethylocta-1,3,6-triene	1186	2.24±0.43	0.26±0.01	0.76±0.02	0.17±0.01	1.95±0.11
1,2,4,5-Tetramethylbenzene	1380	0.44±0.23	0.36±0.16	0.87±0.23	0.89±0.11	0.53±0.12
4,7-Dimethyl-1-propan-2-yl-1,2,3,5,6,8a-hexahydronaphthalene	1692	0.41±0.11	0.26±0.04	0.26±0.02	ND	1.40±0.23
(4*S*)-1-methyl-4-prop-1-en-2-ylcyclohexene	1133	0.99±0.25	ND	ND	ND	0.39±0.03
1,2,3,4-Tetramethyl-5-methylidenecyclopenta-1,3-diene	1433	0.53±0.09	ND	ND	0.79±0.05	ND
1-Methylnaphthalene	1800	ND	ND	0.25±0.10	0.21±0.04	ND
Total miscellaneous		16.35±1.06	13.26±0.98	12.61±1.55	13.02±0.74	18.68±0.84
Total volatiles		134.75±3.01	50.57±3.64	62.60±6.36	65.14±5.32	82.65±2.71

[a] The data were expressed as mean value ± SD; ND: Not detected.

[b] RI: Retention index.

Table S3 The volatile compositions of black teas from different tea cultivars (μg guaiacol equivalent/g dry tea).[a]

Compounds	RI [b]	Chunyu2	Chunyu1	Longjing43	Zhenong117	Jinguanyin
Alcohols						
3,7-Dimethylocta-1,6-dien-3-ol	1524	577.99±45.94	109.92±6.08	103.11±15.16	244.62±26.36	205.52±20.52
(2*E*)-3,7-Dimethylocta-2,6-dien-1-ol	1806	206.51±13.87	182.76±28.73	244.16±23.27	173.47±12.24	879.18±58.63
2-[(2*S*,5*S*)-5-Ethenyl-5-methyloxolan-2-yl]propan-2-ol	1443	108.50±10.88	29.71±5.03	39.75±3.54	31.31±7.43	61.15±8.72
2-[(1*S*)-4-Methylcyclohex-3-en-1-yl]propan-2-ol	1664	19.68±2.43	4.05±0.53	1.47±0.18	5.94±0.87	8.15±1.06
2-Phenylethanol	1838	8.15±0.78	6.17±0.62	6.76±1.01	9.85±2.81	5.44±0.64
Hexan-1-ol	1342	2.66±0.83	2.96±0.75	9.12±0.83	5.38±1.09	3.33±0.77
6-Ethenyl-2,2,6-trimethyloxan-3-ol	1704	15.80±2.68	ND	1.81±0.59	1.52±0.60	ND
(*Z*)-Hex-3-en-1-ol	1370	3.57±0.78	1.68±0.64	4.66±0.21	6.24±1.38	2.20±0.44
(1*S*,2*R*,5*S*,7*R*,8*R*)-2,6,6,8-Tetramethyltricyclo[5.3.1.01,5]undecan-8-ol	2022	2.52±0.26	1.91±0.14	2.09±0.75	2.97±0.59	8.25±1.36
Phenylmethanol	1821	3.48±1.20	2.44±0.24	4.27±0.53	4.31±0.98	2.90±0.23
Nonan-1-ol	1628	1.54±0.22	6.23±0.31	2.64±0.39	3.94±0.45	2.35±0.47
2-Ethylhexan-1-ol	1465	2.07±0.71	1.27±0.25	3.67±1.36	4.78±1.51	4.37±0.29
(6*E*)-3,7,11-Trimethyldodeca-1,6,10-trien-3-ol	1899	ND	ND	3.37±0.76	1.37±0.33	10.67±2.40
(*E*)-Hex-2-en-1-ol	1390	3.20±0.42	3.51±0.67	3.03±0.29	4.96±0.74	ND
Dodecan-1-ol	1868	2.83±0.52	1.65±0.30	1.69±0.54	1.57±0.36	6.73±0.66
(6,6-Dimethyl-2-bicyclo[3.1.1]hept-2-enyl)methanol	1752	ND	8.44±1.32	ND	ND	ND
(5*E*)-3,7-Dimethylocta-1,5,7-trien-3-ol	1582	2.62±0.49	0.79±0.07	0.26±0.04	2.20±0.33	2.44±0.27
Octan-1-ol	1533	ND	ND	3.38±1.56	3.55±0.84	ND
(*Z*)-Non-3-en-1-ol	1650	0.78±0.27	1.38±0.10	1.29±0.21	1.33±0.12	ND
(1*S*,4*R*)-1,6-Dimethyl-4-propan-2-yl-3,4,4a,7,8,8a-hexahydro-2H-naphthalen-1-ol	2044	0.85±0.20	0.47±0.02	ND	0.66±0.29	1.95±0.30
Heptan-2-ol	1310	3.06±1.24	ND	ND	ND	ND
(3*Z*)-3,7-Dimethylocta-3,6-dien-1-ol	1774	ND	ND	1.54±0.14	0.62±0.21	ND
Heptan-1-ol	1436	ND	0.68±0.18	0.49±0.10	0.70±0.05	ND
(3*E*,6*E*)-Nona-3,6-dien-1-ol	1717	ND	ND	0.64±0.19	0.56±0.11	ND
Total alcohols		965.82±62.61	365.99±42.71	439.17±45.51	511.86±44.46	1204.65±78.42
Aldehydes						
2,4-Dimethylbenzaldehyde	1765	56.69±13.80	44.58±6.19	48.96±1.08	46.38±1.61	197.64±18.64
(2*E*)-3,7-Dimethylocta-2,6-dienal	1690	15.44±2.12	ND	21.30±2.28	10.14±0.99	54.96±4.15

(2*Z*)-3,7-Dimethylocta-2,6-dienal	1643	5.30±0.24	16.04±2.33	8.35±1.04	3.72±0.53	14.91±0.86
(*E*)-Hex-2-enal	1189	ND	8.16±2.24	8.72±0.82	11.86±1.60	6.89±1.19
Hexanal	1044	3.46±0.43	3.50±0.91	4.58±0.54	5.41±1.00	3.89±0.60
Nonanal	1348	2.19±0.36	2.19±0.78	2.20±0.31	2.12±0.84	8.68±2.22
3-Methylbutanal	936	3.55±0.80	2.33±0.82	6.87±0.52	3.43±0.48	ND
Benzaldehyde	1487	4.58±0.80	2.09±0.26	3.56±0.78	2.27±0.05	2.29±0.33
(4*E*,8*E*)-5,9,13-Trimethyltetradeca-4,8,12-trienal	2066	1.02±0.07	ND	ND	0.98±0.27	12.57±2.00
Decanal	1452	ND	0.87±0.15	ND	ND	4.10±1.20
(2*E*,4*E*)-Hepta-2,4-dienal	1439	0.96±0.18	1.72±0.43	ND	2.00±0.24	ND
(*E*)-Oct-2-enal	1396	1.61±0.53	1.19±0.27	0.53±0.14	1.16±0.12	ND
7,7-Dimethoxyheptanal	1585	ND	0.42±0.02	0.64±0.25	0.52±0.09	2.12±0.61
2,6,6-Trimethylcyclohexene-1-carbaldehyde	1572	ND	1.43±0.82	ND	0.87±0.20	ND
Pentanal	961	ND	0.36±0.11	0.27±0.09	0.35±0.12	ND
(*Z*)-Hept-2-enal	1289	ND	0.44±0.14	ND	0.51±0.09	ND
(*E*)-Non-2-enal	1497	ND	0.42±0.05	ND	0.28±0.03	ND
(2*E*,6*Z*)-Nona-2,6-dienal	1552	ND	ND	0.29±0.04	0.37±0.06	ND
Total aldehydes		94.79±15.21	85.75±12.75	106.27±6.77	92.38±6.31	308.03±18.18
Ketones						
6-Methylhept-5-en-2-one	1311	ND	1.22±0.24	3.08±0.29	1.11±0.18	3.77±0.77
3-Methyl-2-[(*Z*)-pent-2-enyl]cyclopent-2-en-1-one	1850	ND	2.33±0.15	1.34±0.35	5.45±1.30	ND
(*E*)-4-(2,6,6-Trimethylcyclohexen-1-yl)but-3-en-2-one	1846	1.58±0.85	1.87±0.20	3.18±0.60	1.87±0.34	ND
6,10,14-Trimethylpentadecan-2-one	2027	1.27±0.14	ND	0.64±0.09	1.22±0.19	ND
Heptadecan-2-one	1891	ND	0.45±0.04	0.23±0.04	ND	ND
Octane-2,3-dione	1290	ND	ND	0.53±0.08	ND	ND
Total ketones		2.84±0.98	5.86±0.50	9.01±1.14	9.65±0.71	3.77±0.77
Esters						
Methyl 2-hydroxybenzoate	1727	44.92±5.46	45.20±6.95	54.81±7.81	75.57±6.59	63.89±9.22
Bis(2-methylpropyl) benzene-1,2-dicarboxylate	2118	10.48±2.95	4.84±1.16	6.94±1.71	5.12±0.20	26.35±5.25
Diethyl benzene-1,2-dicarboxylate	2084	3.58±1.84	5.08±2.22	2.59±0.05	ND	24.92±8.43
1-*O*-(2-Methylpropyl) 4-*O*-propan-2-yl 2,2-dimethyl-3-propan-2-ylbutanedioate	1817	ND	3.73±0.38	2.06±1.59	3.42±0.55	7.22±1.81
Methyl hexadecanoate	2051	4.01±0.76	2.19±0.52	0.89±0.46	1.23±0.23	6.29±2.13
Dibutyl benzene-1,2-dicarboxylate	2033	ND	ND	2.99±0.46	ND	ND
Methyl 2-methyl-3-oxobutanoate	1669	ND	ND	0.81±0.24	0.81±0.05	ND
Total esters		62.99±7.38	61.03±7.51	71.1±8.99	86.15±5.80	128.68±3.88

Acids						
Dodecanoic acid	2104	ND	2.22±0.89	2.02±0.39	2.75±0.38	20.70±1.39
Octadecanoic acid	2064	ND	ND	ND	ND	6.07±1.17
Nonanoic acid	2040	ND	ND	1.23±0.39	2.18±0.27	ND
Total acids		ND	2.22±0.89	3.25±0.39	4.93±0.32	26.77±2.55
Pyrroles						
1-Ethylpyrrole-2-carbaldehyde	1571	ND	ND	3.42±1.21	ND	ND
Miscellaneous						
7-Methyl-3-methylideneocta-1,6-diene		27.02±4.25	17.29±1.36	22.52±3.77	18.61±2.73	76.74±13.34
Naphthalene	1114	19.93±8.50	19.79±1.14	10.02±1.85	9.69±1.15	51.55±6.86
(3*E*)-3,7-Dimethylocta-1,3,6-triene	1684	19.78±3.47	13.25±1.84	18.48±3.46	15.37±1.72	84.69±17.30
2,4-Ditert-butylphenol	1186	23.02±2.00	15.68±1.42	14.96±3.12	ND	ND
Methylsulfanylmethane	2071	12.42±2.14	8.41±0.60	9.67±1.52	6.43±2.11	4.05±0.68
Methyl (*Z*)-N-hydroxybenzenecarboximidate	899	10.53±1.77	ND	ND	1.87±0.27	6.69±0.70
(4*S*)-1-Methyl-4-prop-1-en-2-ylcyclohexene	1750	8.39±1.91	2.36±0.53	2.66±0.60	3.37±0.61	ND
1,2,4,5-Tetramethylbenzene	1133	3.35±0.78	ND	1.62±0.71	1.46±0.06	9.34±0.32
1,2,3,4-Tetramethyl-5-methylidenecyclopenta-1,3-diene	1380	ND	1.81±0.40	0.41±0.07	1.87±0.13	ND
(1*S*,6*R*)-3,7,7-Trimethylbicyclo[4.1.0]hept-2-ene	1433	ND	0.53±0.03	ND	ND	2.06±0.44
2-Methyl-5-propan-2-ylcyclohexa-1,3-diene	1122	0.67±0.21	0.22±0.06	0.32±0.05	0.25±0.04	1.06±0.18
Total miscellaneous	1112	125.12±8.73	79.34±4.24	80.65±12.66	58.92±5.38	236.17±27.85
Total volatiles		1251.56±70.55	600.20±64.47	712.87±74.63	763.88±58.38	1908.05±86.87

[a] The data were expressed as mean value ± SD; ND: Not detected.

[b] RI: Retention index.

Table S4 The volatile compositions of freeze dried teas from different tea cultivars (μg guaiacol equivalent/g dry tea).[a]

Compounds	RI [b]	Chunyu2	Chunyu1	Longjing43	Zhenong117	Jinguanyin
Alcohols						
3,7-Dimethylocta-1,6-dien-3-ol	1524	1224.71±95.67	286.63±12.32	393.09±22.07	675.84±85.95	260.00±11.04
(2*E*)-3,7-dimethylocta-2,6-dien-1-ol	1806	441.89±28.02	404.07±23.81	817.37±20.12	363.11±49.35	755.01±56.86
2-[(2*S*,5*S*)-5-Ethenyl-5-methyloxolan-2-yl]propan-2-ol	1443	86.26±8.06	34.89±2.11	69.78±2.71	27.87±6.84	37.96±4.13
Nonan-1-ol	1628	8.00±0.57	19.57±1.28	13.62±1.84	13.27±1.89	4.12±0.37
2-[(1*S*)-4-Methylcyclohex-3-en-1-yl]propan-2-ol	1664	32.65±2.65	7.43±0.09	ND	8.02±1.78	6.02±1.41
Dodecan-1-ol	1868	13.87±3.03	5.34±1.08	13.13±2.76	4.80±1.99	8.66±0.86
(1*S*,2*R*,5*S*,7*R*,8*R*)-2,6,6,8-Tetramethyltricyclo[5.3.1.01,5]undecan-8-ol	2022	8.81±0.63	5.53±0.23	14.16±3.65	6.21±1.84	8.18±1.80
2-Ethylhexan-1-ol	1465	4.14±0.34	5.57±1.66	12.92±1.06	3.87±0.10	1.70±0.37
Octan-1-ol	1533	ND	8.52±1.54	14.39±2.86	ND	3.76±1.00
2-Phenylethanol	1838	3.74±0.16	4.86±0.71	7.75±0.75	5.84±0.29	2.92±0.18
(*Z*)-Hex-3-en-1-ol	1370	4.72±1.38	ND	8.05±0.86	4.06±1.00	1.78±0.29
(6*E*)-3,7,11-Trimethyldodeca-1,6,10-trien-3-ol	1899	ND	ND	ND	ND	17.75±2.71
Tetradecan-1-ol	2041	8.07±1.42	ND	5.74±1.51	ND	3.78±0.37
Heptan-1-ol	1436	ND	4.27±0.06	4.14±0.22	3.44±0.59	ND
Heptan-2-ol	1310	9.30±1.29	ND	ND	ND	ND
Hexan-1-ol	1342	ND	1.95±0.54	4.99±0.06	1.71±0.48	ND
Oct-1-en-3-ol	1428	ND	1.42±0.04	3.89±0.87	1.14±0.44	1.26±0.31
Phenylmethanol	1821	ND	ND	ND	3.20±0.48	2.77±0.05
(3*Z*)-3,7-Dimethylocta-3,6-dien-1-ol	1774	1.59±0.17	ND	2.74±0.49	ND	ND
(1*S*,4*R*)-1,6-Dimethyl-4-propan-2-yl-3,4,4a,7,8,8a-hexahydro-2H-naphthalen-1-ol	2044	ND	0.9±0.07	ND	ND	3.31±1.00
(5*E*)-3,7-Dimethylocta-1,5,7-trien-3-ol	1582	1.33±0.22	ND	ND	1.22±0.34	ND
Total alcohols		1849.07±140.09	790.95±33.47	1385.77±39.62	1123.62±144.65	1118.96±64.40
Aldehydes						
2,4-Dimethylbenzaldehyde	1765	245.54±15.53	163.45±16.05	280.91±19.62	281.85±30.53	149.01±16.77
(*E*)-hex-2-enal	1189	264.38±31.47	110.9±2.08	292.51±24.96	201.02±25.62	36.54±3.23
Hexanal	1044	18.40±1.59	13.20±0.20	95.67±7.55	29.20±1.26	7.33±1.28
(2*E*)-3,7-Dimethylocta-2,6-dienal	1690	17.24±1.84	14.21±2.18	50.91±3.09	15.48±0.71	38.18±4.47
Nonanal	1348	10.90±2.00	6.85±1.64	11.92±1.82	8.53±1.57	7.00±1.74
(2*E*,4*E*)-Hepta-2,4-dienal	1439	6.33±0.52	3.48±0.19	9.71±1.30	7.93±2.12	3.18±0.71

(4*E*,8*E*)-5,9,13-Trimethyltetradeca-4,8,12-trienal	2066	7.08±1.20	4.32±0.66	3.61±1.82	5.37±0.22	6.52±0.95
(2*Z*)-3,7-Dimethylocta-2,6-dienal	1643	1.32±0.17	1.20±0.01	6.69±0.94	0.97±0.23	7.90±0.87
Benzaldehyde	1487	3.01±0.23	1.61±0.14	5.30±0.68	2.68±0.19	2.77±0.09
Decanal	1452	4.15±1.35	2.61±0.68	ND	ND	7.59±1.69
(2*E*,6*Z*)-Nona-2,6-dienal	1552	3.67±0.26	1.43±0.25	4.82±0.22	3.99±0.93	ND
7,7-Dimethoxyheptanal	1585	ND	4.32±1.01	4.88±0.36	4.08±0.66	ND
Octadecanal	1894	ND	ND	4.17±0.73	ND	5.70±5.73
(*E*)-Oct-2-enal	1396	1.89±0.07	1.47±0.22	3.44±0.43	1.84±0.54	ND
Heptanal	1141	ND	ND	ND	ND	4.44±0.95
(*E*)-Non-2-enal	1497	ND	ND	2.46±0.30	1.78±0.30	ND
Total aldehydes		583.89±52.05	329.07±18.39	776.97±49.00	564.73±34.33	276.15±26.11
Ketones						
3-Methyl-2-[(*Z*)-pent-2-enyl]cyclopent-2-en-1-one	1850	4.97±0.57	6.69±0.82	6.02±0.34	14.91±1.33	8.40±1.44
(3*E*,5*E*)-Octa-3,5-dien-2-one	1492	ND	ND	10.97±0.87	7.31±1.00	ND
(*E*)-4-(2,6,6-Trimethylcyclohexen-1-yl)but-3-en-2-one	1846	4.48±0.53	ND	6.03±0.57	2.82±0.27	ND
6,10,14-Trimethylpentadecan-2-one	2027	7.88±1.67	ND	ND	ND	3.71±1.73
6-Methylhept-5-en-2-one	1311	1.81±0.17	0.87±0.12	2.07±0.57	ND	1.92±0.21
Heptadecan-2-one	1891	ND	ND	2.25±0.33	ND	ND
Total ketones		19.15±1.54	7.56±0.83	27.33±0.92	25.04±2.59	14.03±2.12
Esters						
Methyl 2-hydroxybenzoate	1727	15.32±2.25	36.83±2.94	71.10±5.86	57.12±5.04	23.95±1.29
Bis(2-methylpropyl) benzene-1,2-dicarboxylate	2118	57.69±8.02	22.61±4.23	49.10±13.00	28.82±7.28	32.48±3.82
[2,2,4-Trimethyl-3-(2-methylpropanoyloxy)pentyl] 2-methylpropanoate	1818	35.51±10.04	24.51±4.70	60.42±10.19	16.56±2.60	22.74±3.32
Dibutyl benzene-1,2-dicarboxylate	2033	ND	16.24±3.71	20.67±4.74	ND	22.32±0.69
(3-Hydroxy-2,4,4-trimethylpentyl) 2-methylpropanoate	1815	12.32±0.56	ND	30.59±6.68	ND	10.46±1.81
Methyl hexadecanoate	2051	8.67±2.09	ND	8.04±0.62	6.54±2.29	7.58±2.39
2-Ethylhexyl 2-hydroxybenzoate	2069	5.40±1.79	ND	10.05±0.89	ND	2.99±0.17
[(*Z*)-Hex-3-enyl] acetate	1279	2.96±0.10	ND	ND	7.68±1.72	ND
Propan-2-yl tetradecanoate	1897	ND	ND	5.94±1.06	ND	3.94±2.61
Total esters		137.87±17.55	100.19±8.66	255.91±33.03	116.71±13.55	126.47±10.75
Acid						
Dodecanoic acid	2104	12.58±1.67	4.92±2.02	13.32±2.95	11.66±3.69	8.68±1.71
Miscellaneous						
7-Methyl-3-methylideneocta-1,6-diene	1114	73.78±7.86	36.9±5.09	106.92±2.69	57.29±8.58	84.15±14.29

2,4-Ditert-butylphenol	2071	77.34±8.88	72.5±2.78	92.61±19.22	66.95±10.24	53.89±6.99
Naphthalene	1684	42.20±1.80	28.59±2.88	63.03±8.33	55.74±4.30	38.59±4.67
(3*E*)-3,7-Dimethylocta-1,3,6-triene (β-ocimene)	1186	38.25±4.00	19.73±3.97	51.93±1.42	23.22±3.39	47.43±10.92
(3*E*)-3,7-Dimethylocta-1,3,6-triene (*trans*-β-ocimene)	1170	22.83±2.58	11.48±1.22	32.21±1.45	14.37±1.91	36.72±3.83
(4*S*)-1-Methyl-4-prop-1-en-2-ylcyclohexene	1133	20.52±0.74	ND	14.07±1.47	12.14±1.23	11.55±2.31
Methyl (*Z*)-N-hydroxybenzenecarboximidate	1750	9.96±1.22	ND	10.29±1.32	9.06±2.12	11.07±2.88
1,2,4,5-Tetramethylbenzene	1380	6.81±1.29	4.12±0.38	8.68±1.38	6.65±1.45	5.48±1.01
4,7-Dimethyl-1-propan-2-yl-1,2,3,5,6,8a-hexahydronaphthalene	1692	5.00±0.49	3.14±0.16	3.88±0.36	ND	14.44±2.28
4-Methylidene-1-propan-2-ylbicyclo[3.1.0]hexane	1143	5.24±1.22	ND	5.30±0.85	ND	ND
(1*S*,6*R*)-3,7,7-Trimethylbicyclo[4.1.0]hept-2-ene	1122	ND	ND	2.72±0.09	1.82±0.52	2.71±0.63
2-Methyl-5-propan-2-ylcyclohexa-1,3-diene	1112	1.73±0.27	0.51±0.09	ND	0.69±0.14	1.28±0.23
Methylsulfanylmethane	899	ND	ND	3.86±0.27	ND	ND
Total miscellaneous		303.67±24.86	176.96±10.85	395.50±23.89	247.93±15.88	307.32±33.30
Total volatiles		2906.23±232.81	1409.65±63.75	2854.8±114.86	2089.68±161.87	1851.61±94.50

[a] The data were expressed as mean value ± SD; ND: Not detected.

[b] RI: Retention index.

Table S5 The IUPAC name list for the symbols in Fig. 6

IUPAC name	Freeze dried tea	Green tea	Black tea
Alcohol			
3,7-Dimethylocta-1,6-dien-3-ol	Fr-Alcohol-1	G-Alcohol-1	R-Alcohol-1
(2*E*)-3,7-Dimethylocta-2,6-dien-1-ol	Fr-Alcohol-2	G-Alcohol-2	R-Alcohol-2
2-[(2*S*,5*S*)-5-Ethenyl-5-methyloxolan-2-yl]propan-2-ol	Fr-Alcohol-3	G-Alcohol-3	R-Alcohol-3
(*Z*)-Hex-3-en-1-ol	Fr-Alcohol-4	G-Alcohol-4	R-Alcohol-4
2-[(1*S*)-4-Methylcyclohex-3-en-1-yl]propan-2-ol	Fr-Alcohol-5	G-Alcohol-5	R-Alcohol-5
Dodecan-1-ol	Fr-Alcohol-6	G-Alcohol-6	R-Alcohol-6
2-Phenylethanol	Fr-Alcohol-7	G-Alcohol-7	R-Alcohol-7
Hexan-1-ol	Fr-Alcohol-8	G-Alcohol-8	R-Alcohol-8
2-Ethylhexan-1-ol	Fr-Alcohol-9	G-Alcohol-9	R-Alcohol-9
(5*E*)-3,7-Dimethylocta-1,5,7-trien-3-ol	Fr-Alcohol-10	G-Alcohol-10	R-Alcohol-10
Nonan-1-ol	Fr-Alcohol-11	G-Alcohol-11	R-Alcohol-11
Aldehyde			
2,4-Dimethylbenzaldehyde	Fr-Aldehyde-1	G-Aldehyde-1	R-Aldehyde-1
(2*E*)-3,7-Dimethylocta-2,6-dienal	Fr-Aldehyde-2	G-Aldehyde-2	R-Aldehyde-2
(2*Z*)-3,7-Dimethylocta-2,6-dienal	Fr-Aldehyde-3	G-Aldehyde-3	R-Aldehyde-3
Pentanal	Fr-Aldehyde-4	G-Aldehyde-4	R-Aldehyde-4
Hexanal	Fr-Aldehyde-5	G-Aldehyde-5	R-Aldehyde-5
Benzaldehyde	Fr-Aldehyde-6	G-Aldehyde-6	R-Aldehyde-6
(2*E*)-3,7-Dimethylocta-2,6-dienal	Fr-Aldehyde-7	G-Aldehyde-7	R-Aldehyde-7
Ester			
Bis(2-methylpropyl) benzene-1,2-dicarboxylate	Fr-Ester-1	G-Ester-1	R-Ester-1
Diethyl benzene-1,2-dicarboxylate	Fr-Ester-2	G-Ester-2	R-Ester-2
[(*Z*)-Hex-3-enyl] acetate	Fr-Ester-3	G-Ester-3	R-Ester-3
Methyl 2-hydroxybenzoate	Fr-Ester-4	G-Ester-4	R-Ester-4
Ketone			
(*E*)-4-(2,6,6-Trimethylcyclohexen-1-yl)but-3-en-2-one	Fr-Ketone-1	G-Ketone-1	R-Ketone-1
3-Methyl-2-[(*Z*)-pent-2-enyl]cyclopent-2-en-1-one	Fr-Ketone-2	G-Ketone-2	R-Ketone-2
Miscellaneous			
(3*E*)-3,7-Dimethylocta-1,3,6-triene	Fr-Miscellaneous-1	G-Miscellaneous-1	R-Miscellaneous-1
Naphthalene	Fr-Miscellaneous-2	G-Miscellaneous-2	R-Miscellaneous-2
2,4-Ditert-butylphenol	Fr-Miscellaneous-3	G-Miscellaneous-3	R-Miscellaneous-3

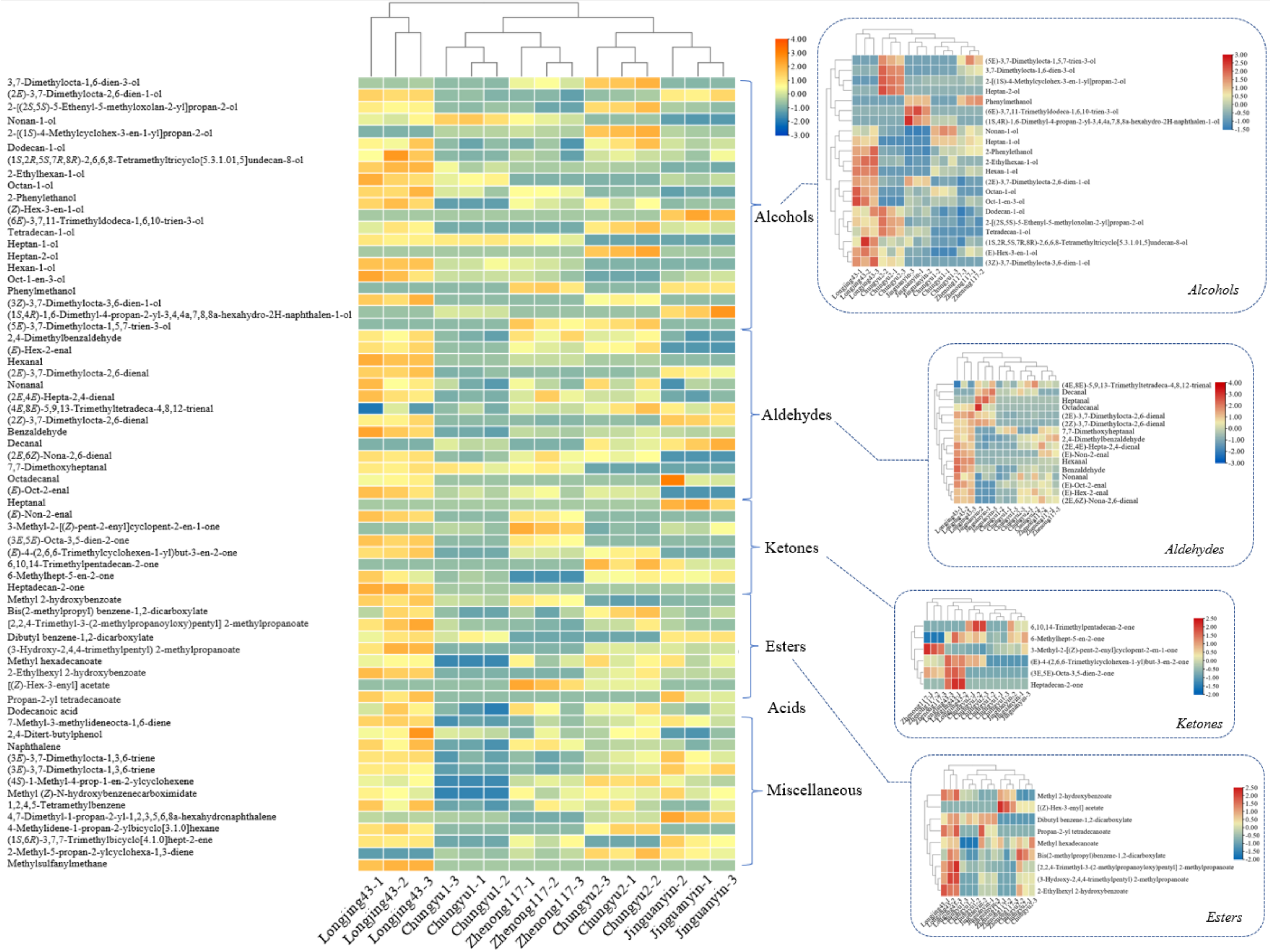

Figure S1. The hierarchically heatmap of the relative contents of volatile compounds in different freeze dried samples. Guaiacol was used as an internal standard to normalize the metabolite signal.

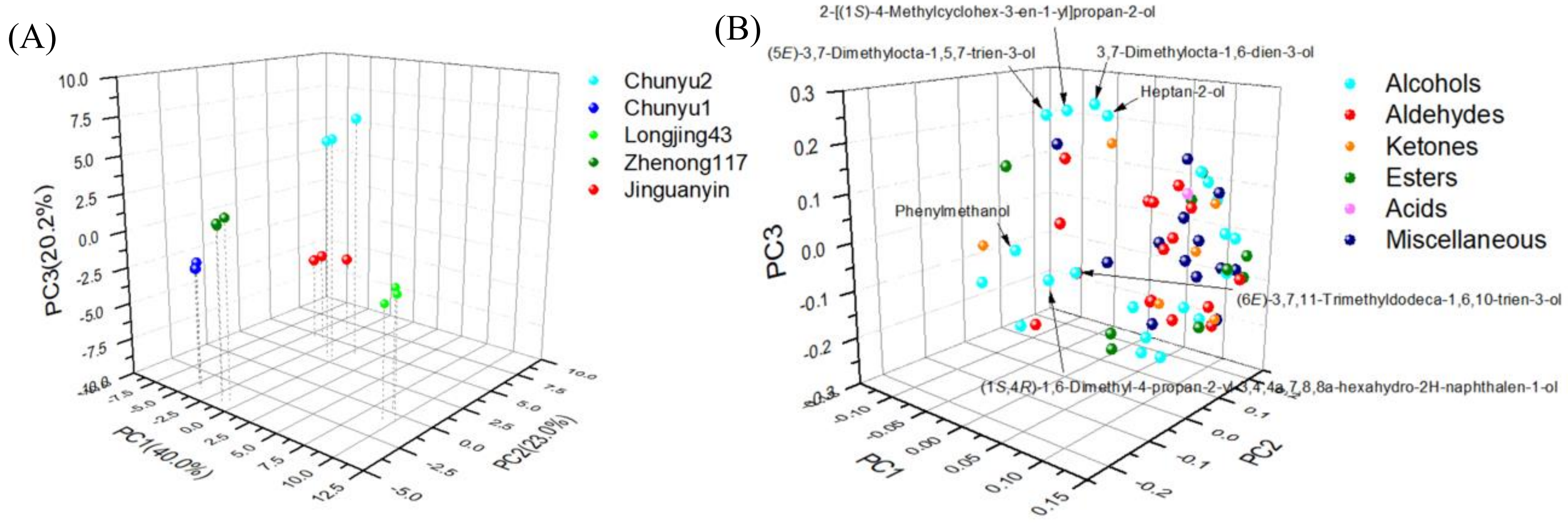

Figure S2. The PCA result of freeze dried samples of different tea cultivars based on the volatile compositions. (A) score plot; (B) loading plot. The number of replicates is equal to 3.
